# Group Poetry and Quilt‐Making –Creative Public Involvement Approaches to Contentious Concepts in Frailty Research

**DOI:** 10.1111/hex.70872

**Published:** 2026-09-14

**Authors:** William Lammons, Lily Arnold, Sarah Mcmullen, Fiona Aspinal, Samina Begum, Katherine Barrett, Kate Walters, Andrew Clegg, Stuart Mackay‐Thomas, Sara Humphrey, Danielle Nimmons

**Affiliations:** ^1^ Department of Primary Care and Population Health University College London (UCL), UCL London UK; ^2^ Keele University, School of Medicine Keele UK; ^3^ Academic Unit for Ageing & Stroke Research University of Leeds, Bradford Teaching Hospitals NHS Foundation Trust Bradford UK; ^4^ Hampstead Group Practice London UK; ^5^ Westcliffe Health Innovations Ltd, Eccleshill Treatment Centre Bradford UK; ^6^ University of Leeds Leeds UK

**Keywords:** creative, frailty, patient and public involvement

## Abstract

**Introduction:**

‘Frailty’ as a concept is being increasingly integrated into healthcare, but the language around frailty is contentious and potentially stigmatising to patients. It is therefore important to understand how patients’ lived experiences relate to the concept of frailty. We conducted 3 creative patient and public involvement (PPI) workshops to support the study, ‘Exploring how the Electronic Frailty Index 2 (eFI2) can be best utilised in clinical practice.’ Our aims for these workshops were to 1) improve our eFI2 project topic guide and data analysis 2) explore whether creative PPI can support engagement with contentious concepts like frailty, and 3) to understand how frailty can be communicated to patients.

**Methods:**

Creative PPI offers a means of addressing the shortcomings and challenges of PPI, including tokenism, accessibility, and inclusivity, by allowing patients and members of the public to contribute in different, individual, and non‐discursive ways. We used group blackout poetry writing and quilt‐making as creative PPI approaches to understand how patients relate to ‘frailty.’ We collaborated with PPI networks affiliated with the NIHR Applied Research Collaborations (ARCs) and a South Asian community organisation to recruit participants and used public collaborators to co‐design and ‐deliver activities. We used qualitative thematic reflections to share findings from these activities.

**Results:**

Twenty‐two people attended three creative PPI workshops (two blackout poetry activities and one quilt‐making activity). These activities provided three types of findings: 1) the impacts of the creative PPI activities on the eFI2 research process, 2) three thematic reflections, ‘difficulties with ‘frailty’ as a term and concept,’ ‘personalising ‘frailty’ in patient communications,’ and ‘time pressures and dysfunction in healthcare services;’ and 3) the creative outputs (text of two poems and an image of the quilt).

**Conclusion:**

Research and healthcare professionals should work towards discussing frailty with patients in terms and concepts that reflect their experiences and understanding and consider their overall health. Future research and PPI should focus on diverse cultural perspectives on frailty.

## 1 Introduction

### 1.1 Frailty and the Electronic Frailty Index 2

While ‘frailty’ is a helpful concept and term for researchers and clinicians, it presents many challenges for patients and members of the public in research and clinical spaces [1, 2]. Frailty is a clinical condition where people experience a loss of reserve and excessive vulnerability to adverse outcomes following minor stressors [1, 2]. Using it as a concept allows researchers and clinicians to understand declines in health and capability in a global and centralised way [1, 2].

However, previous research has noted the negative connotations that the concept ‘frailty’ has for patients [2, 3], and the lack of shared understanding of ‘frailty’ in healthcare settings [4, 5]. This accompanies a tension between celebrating positive aspects of ageing and recognising that some older people have accumulated disadvantage during their lives. Universally positive frameworks and discourses tend to ignore how particular groups of older people carry disadvantage into later life, frequently manifesting as frailty, with negative impacts on wellbeing [6].

Different tools are available to aid in the identification and management of frailty, as a clinically diagnosable condition, including the Electronic Frailty Index 2 (eFI2) a tool recently incorporated into electronic health records and available to primary care clinicians in England [7, 8]. The ‘Exploring how the Electronic Frailty Index 2 (eFI2) can be best utilised in clinical practice’ study collected and analysed qualitative data from focus groups and interviews with 31 primary care healthcare professionals based in England to explore how eFI2 could be utilised in clinical practice [8].

Given the evident differences between clinician and patient views on frailty [9, 10], we held patient and public involvement (PPI) activities to shape creation of the study's qualitative topic guides and guide analysis of responses. We chose to use creative methods in PPI activities as a more inclusive approach to engaging participants on frailty topics with researchers [9, 10].

### 1.2 Creative PPI

This paper focuses on the creative patient and public involvement (PPI) activities for the eFI2 study [8]. The National Institute of Health and Care Research (NIHR) describes PPI as an ‘active partnership’ between the public and the research team which facilitates power‐sharing between parties and collaboratively shapes the research project [11, 12, 13, 14]. Despite the prevalence and successes of PPI, the approach continues to face limitations and gaps attributed to disingenuous involvement or ‘tokenism,’ varied support for patients’ accessibility needs, focusing too heavily on discussion‐based activities, and using technical language over Plain English [15, 16, 17, 18, 19].

Creative methods offer a novel way of expanding PPI's ability to engage and involve people in the research process and address these gaps [17, 18, 19, 20, 21, 22]. One of the greatest strengths of ‘creative PPI’ is that it can shift the collaboration's focus away from speech and discussion to allow patients and members of the public to contribute in different, individual, and non‐discursive ways, in a sense, making topics easier to reflect on and engage with, especially those too abstract or emotionally difficult to discuss exclusively with question and answer approaches [17, 23, 24]. These different ways of collaborating can ‘say the unsayable’ and create space away from technical jargon and academic power dynamics to build more active partnerships, genuine interactions, and relationships with PPI members [17, 21, 23].

Yet, creative PPI still faces unique challenges of its own. First, defining what constitutes ‘creative PPI’ remains blurry and varies between different studies and creative projects [17]. In their systematic review, Philips et al. found that ‘creative PPI’ usually refers to a variety of arts activities (painting, drawing), multimedia (video, animation), creative writing (poetry, creative narratives), drama (improvisation, acting, forum theatre) or media as a stimulus for conversation or discussion (Facebook, photo elicitation, Legos) [17]. However, other terms are frequently used besides ‘creative PPI,’ such as ‘participatory arts‐based approaches,’ or simply ‘creative health’ [17, 20, 21, 25, 26, 27, 28, 29].

Creative PPI's other limitations include unique challenges around time and resources, ethics, the generalisability of insights and outputs, and a lack of structural support or infrastructure for creative processes in research institutions [17]. Phillips et al. point out that artistic productions that emerge from creative PPI are often unique and compelling to individuals but difficult to generalise to other people or other regions, a particular challenge which clashes with conventional scientific principles of reproducibility and objectivity [17].

### 1.3 Aims

In these creative PPI activities, we aimed 1) to improve our eFI2 topic guide and data analysis through PPI, 2) to explore the extent to which creative PPI can help patients and members of the public engage with contentious and polemical concepts and subjects related to frailty, and 3) to understand how frailty can be communicated to patients in primary care.

This paper has followed the GRIPP2 reporting checklist, developed to ensure transparency and consistency in the documentation of PPI work [30].

### 1.4 Statement on Artificial Intelligence

No large language models or artificial intelligence were used in the process of this paper's methods, analysis, discussion, or writing.

## 2 Methods

We integrated PPI into the eFI2 study through three approaches 1) public collaborators, 2) PPI poetry workshops, and 3) a PPI quilting workshop.

As the terms and concepts, ‘frail’ and ‘frailty,’ present challenges for patients, we chose to use group poetry to allow PPI participants to unpack and deconstruct their meanings [17, 21, 23]. Poetry's ability to portray life experiences in individual or community settings offers research a tool to maintain focus on language while exploring differing complex and contested meanings [12, 26, 27, 28].

We chose quilting as an additional and inclusive method to engage participants with limited English language skills and support them in engaging with ‘frailty’ conceptually and with less focus on the actual language around it [17, 21, 23]. Quilting especially allows group members to collectively create ways of knowing and encourages storytelling [31]. Poetry and quilt‐making helped us create spaces where members could work together to convey experiences and thinking while also creating visual representations of them.

### 2.1 Public Collaborators

Two lead PPI members with self‐identified lived experience of frailty (either experiencing frailty themselves or acting as a carer), SB and KB, were ‘public collaborators’ for the eFI2 study and were involved more in‐depth in the project than other workshop participants [32]. They are based in Bradford and London and have collaborated with PPI professionals at the NIHR Applied Research Collaborations (ARCs) North Thames and Yorkshire and Humber, applied health research infrastructures that connect community groups, patients, researchers, and policy [33]. As collaborators, they reviewed the grant application and interview topic guides prior to submission and use. Discussions around these research materials with our public collaborators also led to shaping the eFI2 study in unexpected ways, such as leading us to explore carers’ roles in frailty and diversifying our recruitment to a range of locations across England.

After securing funding, the public collaborators helped design the PPI workshops and continued to act as co‐authors on the study's publications. They also took part in an analysis meeting, where a summary of results was presented and discussed with them so that they could shape data interpretation and the results section of all resulting manuscripts.

### 2.2 Recruitment, Demographics and Payments

Participants for these PPI activities were recruited in multiple phases. First, the authors worked with the NIHR ARC Yorkshire & Humber to recruit four members of the NIHR ARC Yorkshire & Humber's Frailty Oversight Group (FOG) for the Bradford poetry activity. This included one man and three women, all from white backgrounds, well educated, based across Yorkshire, and aged 65+ years old. All had either lived experience of something that could be described as ‘frailty,’ themselves, or had been a carer for someone with frailty. SB, a woman of South Asian background from Yorkshire aged between 50 and 60 years old with experience of caring, served as public collaborator during the Bradford poetry session [7].

The London poetry session attendees were recruited through the NIHR ARC North Thames, a North and East London‐based ARC with multiple PPI panels and recruitment networks [33]. These attendees were recruited through an open call email that was sent to multiple mailing lists grounded in relationships managed at the ARC North Thames. London poetry session attendees represented a more diverse group of four attendees (2 white, 1 Black British Caribbean, 1 South Asian) based in East London, North London, and Essex. Attendees ages ranged from 40 to 60+ and represented a variety of educational backgrounds (bachelor's degree, some university study, no university study). All members had experience of caring, and some had lived experience of what could be described as ‘frailty,’ though they would likely not describe themselves as ‘frail.’

To reach a broader variety of experiences and identities, we recruited for the quilting workshop through a public collaborator who liaised with a local Bradford community organisation, the Khidmat Centre. They explained the research project to a broader audience through the Centre and solicited interest, identifying the greatest interest from an elderly (60+ years old) Urdu‐speaking Pakistani women's group of 14 members who mostly could not read or write in any language and experienced varying levels of deprivation.

All attendees for the activities and were paid by NIHR rates, £25/hour of PPI activity [34]. The authors note that the rates have since increased to roughly £27.50/hour [35]. Lunch and refreshments were provided, and transport fees and caring costs were reimbursed as needed. Public collaborators were paid for their time spent helping organise the activities and for their time spent writing and editing this manuscript.

### 2.3 Poetry Workshops

We applied our understandings of poetry as a tool to understand and express human experience to a group setting, aiming to use poetry as a means of collective storytelling capable of better illustrating lay peoples’ conceptions of the realities that the term ‘frailty’ attempts to represent [36, 37, 38]. These workshops also explored the eFI2 research findings on the meanings of ‘frailty’ and the ways in which it is communicated to patients in clinical settings. These workshops were deliberately spaced throughout the study, ensuring that workshop outcomes could shape the direction of the research process. The first workshop was held in Bradford halfway through data collection to explore if further questions should be added to our topic guide. To minimise costs the session was held in the project's usual location, a meeting room at Bradford Royal Infirmary, a space with no room charges.

The second workshop in London, led by WL and LA, occurred when eFI2 qualitative data collection was finished to prompt and frame analysis processes. Our thinking had developed since the first poetry session, and we sought to expand the diversity of PPI attendees and inclusivity of the activities, so we held the activity in a community centre in Islington, avoiding the negative connotations that universities and hospitals can hold for some members of the public [31]. An additional co‐author attended and helped to facilitate the London session [39].

The Bradford and London activities were held in similar formats with slight differences, shown in Table 1.

**Table 1:** Workshop outlines for both poetry workshops.

Bradford workshop outline:		London workshop outline:
Introductions, lunch provided Blackout poetry activity Initial findings presentation with room for breakout discussion Access break Collective poetry writing and discussion		Introductions, tea and coffee provided Blackout poetry activity Initial findings presentation with room for breakout discussion Access break, Lunch served Ongoing discussion, and start of collective poetry writing Read and discuss the poem written in the Bradford workshop

#### 2.3.1 Blackout Poetry

**Figure 1:**
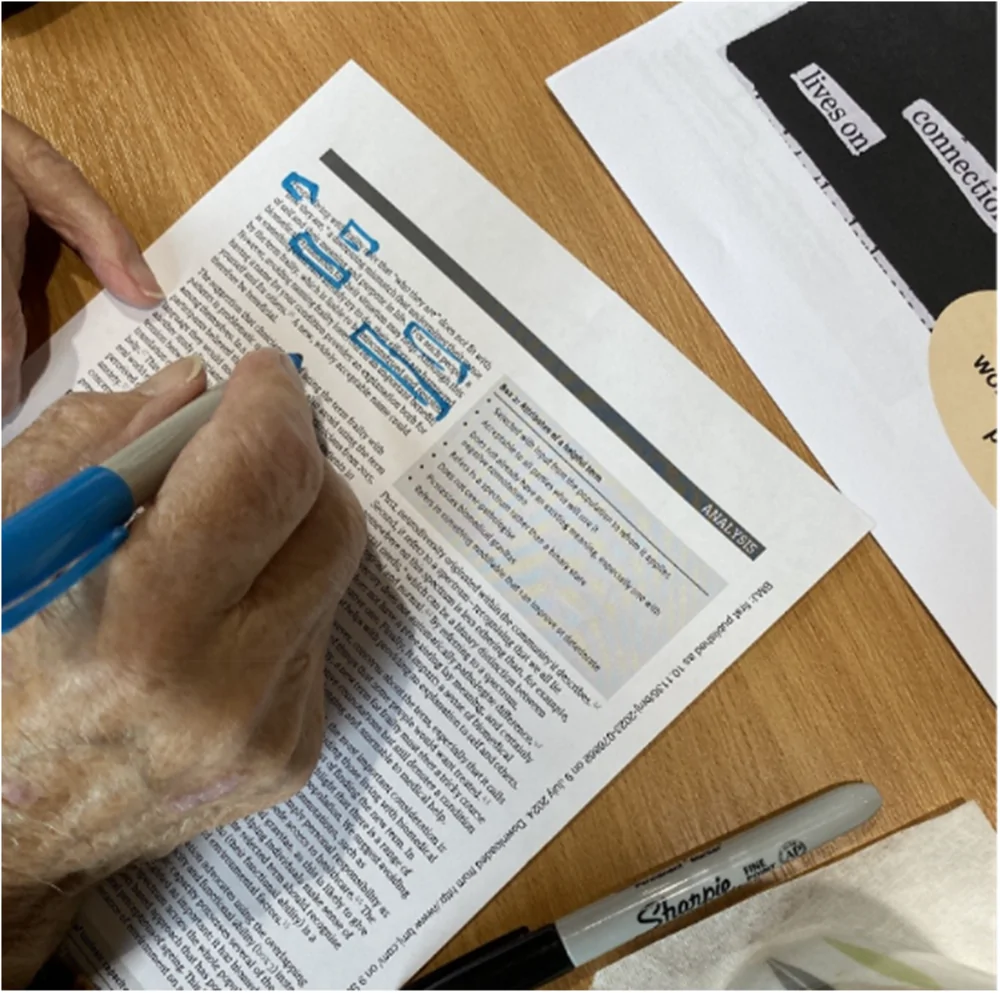
Circling words to create blackout poetry.

**Figure 2:**
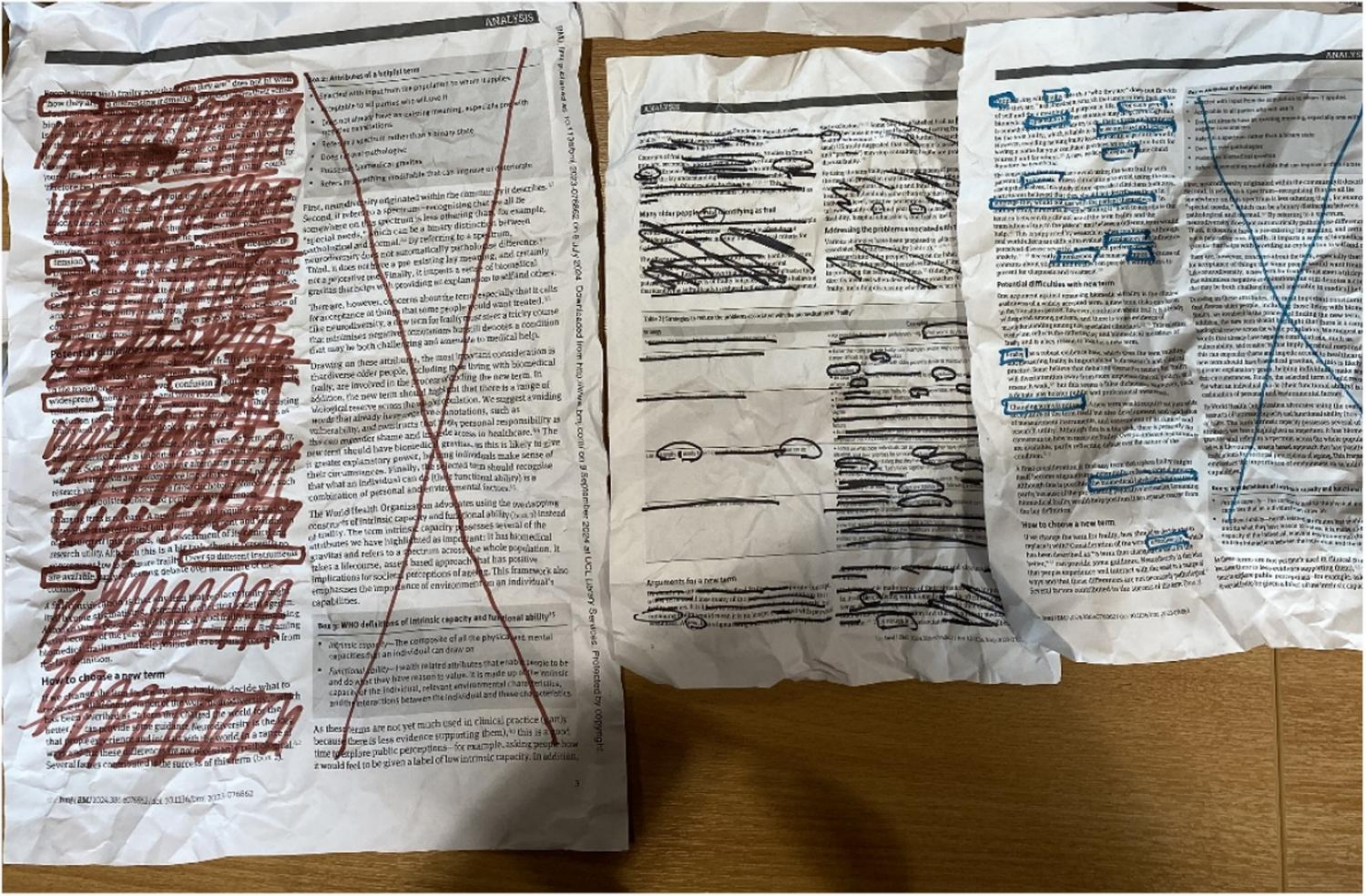
Crossing out words to create blackout poetry.

We began both PPI sessions by creating ‘blackout poetry,’ in which participants make poetry by choosing words or phrases from an existing text (Figure 1) and crossing or ‘blacking out’ those remaining (Figure 2) [37, 38, 40]. We chose Hopkins’ et al.'s, ‘Does frailty need a new name?’ [2], as the session's text, as it examines the tensions around the term ‘frailty’ with limited jargon.

We leaned on the tension that can accompany a sudden call to scribble over the text of an academic journal to inspire participants to challenge the prestige of academic formats and celebrate their own knowledge through their interpretation of the text. This informal method of poetry writing creates a more comfortable and accessible writing process, especially for participants with limited poetry experience.

After 5 min of circling and blacking out words, we asked participants to crumple their poem and toss it to another member of the group, based on Swords et al.'s participatory research technique of ripping up official Newcastle maps to encourage participants to make their own [41]. We then asked participants to read aloud the poem that was thrown to them, reducing the pressure on participants to read their own poem.

These techniques to improve accessibility of the activities were also intended to ease the burden on participants to contribute, improving the extent to which their contributions were their own and not influenced by the facilitators.

#### 2.3.2 Arranging Phrases and Creating Poems

**Figure 3:**
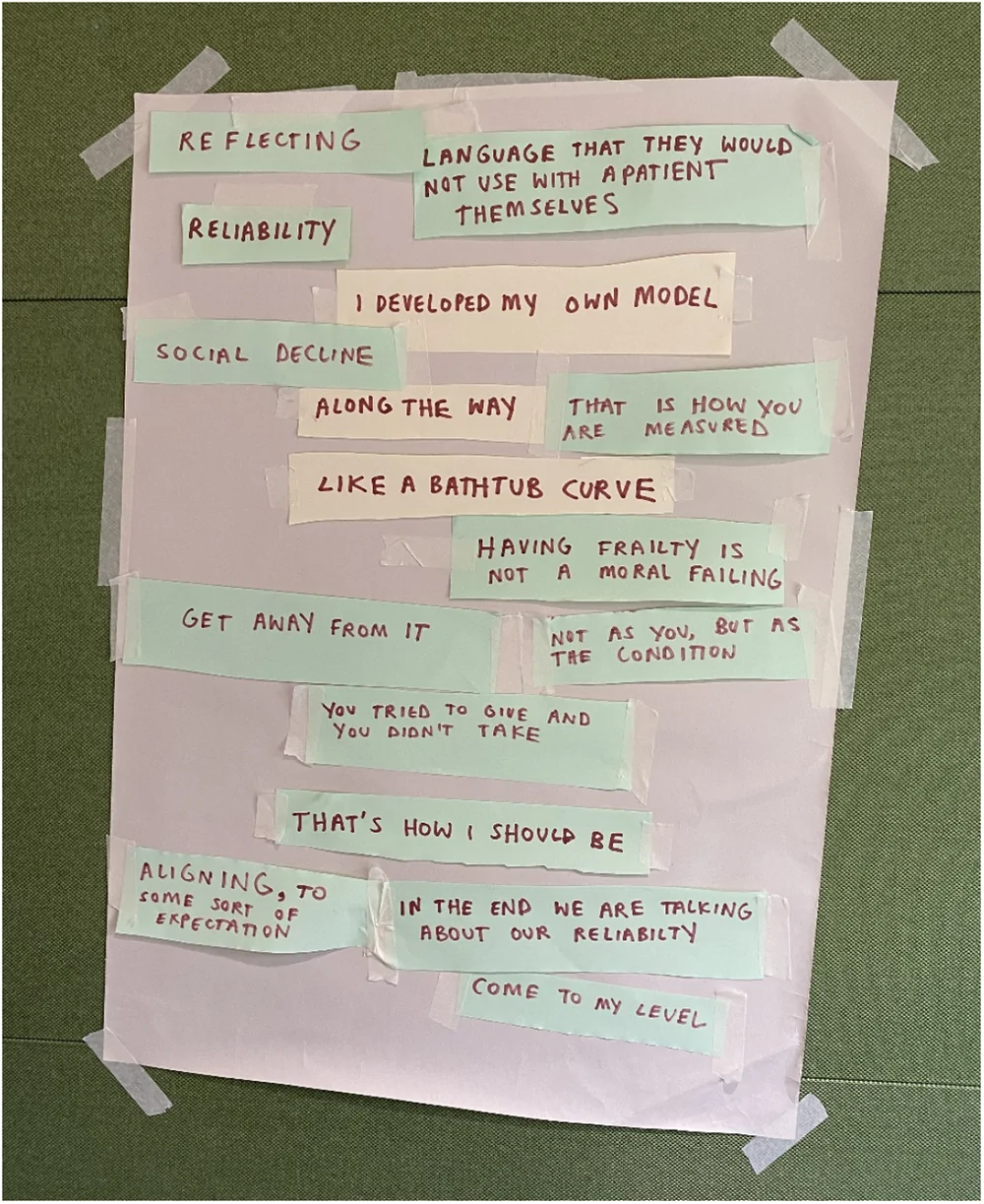
Selected words and phrases from bradford poetry workshop.

Following the blackout poetry activity, we discussed the initial findings of the research project in both the Bradford and London poetry sessions, with space for breakout discussions. A co‐author prompted participants to explore how they wanted healthcare professionals to discuss frailty with them by selecting circled words and phrases to arrange into a poem. LA explained that the poems would be read by those working in primary care and then compiled the phrases as strips of paper on the wall for participants to read and rearrange (Figure 3).

At the workshops’ conclusions, participants reflected on the words chosen for the poems and how they compared to their discussions. To mitigate facilitator biases on the activities, LA wrote and shared a workshop summary which covered key discussion points and a copy of the poem with participants to allow them to correct or edit any elements. No participant raised an issue or correction.

### 2.4 Quilting Workshop

After working with the Bradford FOG for the first poetry session, the research team and public collaborators discussed our need to collaborate with those experiencing frailty who also had experienced socio‐economic deprivation and limited English language abilities. We had initially only planned two Yorkshire poetry workshops, and an additional internal bursary co‐produced with our public collaborator offered an opportunity to expand this PPI work.

SB recommended we work with their local community centre, the Khidmat Centre, a charity providing health and wellbeing support and services for ‘vulnerable members of the community‐the elderly, people with learning disabilities, women, young people and more recently women leaving the criminal justice system,’ [42]. Based on SB's feedback, we chose to change the second workshop to suit this group's needs and move away from the language‐centric focus of poetry. We opted for quilting as an alternative to poetry, based on its long history of representing social experiences, especially by women [43, 44, 45], and allowing participants to collectively create ways of knowing that disrupt linear and individual story telling [46]. In our case, the activity of quilting and the slow pace of hand‐stitching also opened space for discussing frailty.

LA spent considerable time prior to the Bradford quilting session building trust with potential PPI attendees. SB also spent extensive time with the Khidmat Centre, attending the women's group for months in advance to build trust with attendees. Most of the attendees spoke English as a second language, creating a language barrier between them and LA, who does not speak Urdu. SB and the community worker, who both speak Urdu, translated between LA and the group members during both sessions.

This helped those with limited English skills to participate more inclusively in the activity, but it presented challenges to documenting the conversations. LA managed these challenges by checking her understanding, offering opportunities for clarifications, stimulating and supporting discussion, and focusing on creating the quilt as the chief output. It also introduced the potential for biases on the part of the translators, since LA and the research team could not validate or analyse the participants’ comments in their original language. To address this, we relied on our relationships and trust in our public collaborators and contacts at the Khidmat Centre who could speak Urdu to assure a balance of quality, equity, and faithfulness in their translations. We spoke English, when possible, to clarify things and build relationships with the participants during the quilting activities, and we expanded the activity to take place across two workshop meetings to give more time for engagement and relationship‐building (Table 2).

**Table 2:** Workshop outlines for two quilting workshops.

First quilting workshop	Second quilting workshop
Watched a video of a healthcare professional explain frailty in Urdu Discussion about how the attendees understood frailty related to our research findings Explanation of quilting workshop Quilting and discussion about frailty Lunch served from community centre catering service	Tea and biscuits together Quilting and discussion about frailty

In the first quilting workshop, participants watched a video about frailty in Urdu, after which LA and SB held a short discussion structured around some of our initial research findings from the eFI2 study, particularly about the meaning of frailty. Participants began working together on quilt patches following this discussion and sharing of findings. Lunch was then eaten together, prepared by the community centre's catering service. At the end of the first workshop, the group wanted to keep working on their patches, so LA offered to return for an unplanned second session. In the second session, patches were connected into a quilt which was given to the community group for them to keep (Table 2). The finished Bradford quilt became a tactile archive, holding the stories of those who contributed to the project, and was gifted to the community centre upon completion. Following the workshops, LA and SB spent time providing additional aftercare and debriefing for attendees, given the emotional difficulty of discussing frailty.

### 2.5 Analysis

Building on Owrid et al., we have chosen to not use a single or formal analysis methodology [19] for two reasons. First, like Owrid et al., we have chosen to present more accessible findings and results, in concordance with our decision to use creative PPI to have more genuine and accessible discussions and engagements around frailty [19]. We aim to avoid a ‘co‐opting’ of these more accessible creative outputs and activities by a more conventional research agenda or approach [19]. Second, the variety of insights and things that could be described as ‘data’ generated from our creative PPI process do not easily fit a conventional qualitative analysis process [47], including, creative pieces, meetings‐based feedback on PPI activity and project designs, activity and meeting notes, and quotations handwritten by facilitators. To work meaningfully with the insights afforded by all of these elements, we have opted for a multi‐pronged qualitative‐based approach to engage with the insights around our PPI activities in which we can 1) share and reflect on the impacts of these activities on the eFI2 project, 2) complete thematic reflections on the activities and their processes as a whole, and 3) share and reflect the creative productions, themselves [48].

DM, LA, and WL took turns reviewing and reflecting on all data, including transcripts, notes, summary reports, creative pieces, and impact logs to build thematic reflections in narrative formats which represented a complex variety of arguments and perspectives across all activities [49, 50]. As our focus in this project was on the creation of creative outputs and supporting spaces for collaborative reflection around a complex and polemical topic, we did not aim to achieve ‘data saturation,’ or the point at which no new insights can be identified [51, 52].

We present these poems and images of the quilt to preserve their creators’ voices and allow readers to have their own subjective experience with the creative work [22, 38, 53]. Our analysis builds on ideas in visual anthropology and documentary films and relies on the idea that sharing creative works from people with lived experiences of frailty can introduce these experiences to others ‘by acquaintance’ [54, 55].

Our discussion section includes details on the impacts that these creative PPI activities had on the study, the researchers, and the members of the public. However, our paper's contribution is to challenge the typical expectation that PPI's worth lies in impacts it has on a study, by focussing attention on the unique creative outputs that can, themselves, shape research, thinking, and concepts more broadly.

## 3 Results

We held three creative PPI activities (2 poetry, 1 quilt‐making with two parts) with twenty‐two participants. Eight (n = 4 Bradford, n = 4 London) attended the poetry sessions, and fourteen (Bradford) attended the quilt making session. We share three types of analysis in this section: 1) the impacts the creative PPI activities had on the research process (Table 3); 2) three thematic reflections (‘difficulties with ‘frailty’ as a term and concept,’ ‘personalising ‘frailty’ in patient communications,’ and ‘time pressures and dysfunction in healthcare services’); and 3) the creative outputs (text of two poems and an image of the quilt).

**Table 3:** Project timeline with PPI activities and impacts.

Date	eFI2 primary project activities	PPI activities	PPI impacts on the eFI2 study
May‐July 2023	Writing grant application	Recruited Public Collaborators Public Collaborators review grant application	Following PPI review, we decided to focus on differences of frailty according to different groups, specifically focusing on location (West Yorkshire vs London), different ethnicities and deprivation. We also used feedback to ensure that confidentiality statements were clear.
Sept 2023	Grant approved		
June 2024	Ethical Approval		
July 2024	Participant recruitment		
Aug. 2024	Participant recruitment Data collection	Public collaborators help design PPI workshops	We made edits to discussion guides and activity plans to make the poetry workshops more accessible to public participants.
Sept. 2024	Participant recruitment Data collection		
Oct. 2024	Participant recruitment	Bradford PPI poetry workshop with public collaborator	This helped the research team build rapport with the PPI workshop participants. We added an extra prompt to the topic guide following the workshop, which explored using the patient's own understanding and language around frailty in consultations. We decided to hold an additional third workshop for people from ethnic minority backgrounds, based on our PPI collaborator's feedback. In discussion with our research team and public collaborator, we decided to hold a quilting workshop as an alternative engagement method where participants could express their views without using the English language.
Nov. 2024	Data collection		
Dec. 2024	Participant recruitment	Applying for PPI department bursary with our public collaborator	We obtained funding to run a workshop with people from South Asian backgrounds who spoke Urdu.
Jan 2025	Coding	London PPI poetry workshop with public collaborator	PPI participants’ experiences of frailty communication in primary care echoed our results, including the point that discussions around frailty should focus on mental health and isolation, not exclusively medical issues.
Feb. 2025	Coding Analysis		
March 2025	Coding Analysis	Analysis meeting with public collaborators to review results	Public collaborators affirmed that results resonated with their own experiences, including professionals’ lack of use of the word ‘frail’ in consultations.
Apr. 2025	Analysis Writing up		
May 2025	Analysis Writing up	Bradford quilting workshop with public collaborator	
June 2025‐present	Ongoing writing up and analysis		

### 3.1 eFI2 Project Impacts and PPI Decisions

In Table 3, we illustrate how these activities accompanied our regular project timeline and the actions we took in response.

The topic guide was modified in response to PPI workshop discussions, especially the section that posed questions about professionals’ ways of communicating ‘frailty’ to patients. We added additional prompts to our eFI2 study topic guide to explore whether participants had experience using the patient's own words and understanding to explain frailty. We also included a broader range of professionals in eFI2 data collection to specifically get a better understanding of frailty by location, ethnicity, and socio‐economic deprivation and improved its overall clarity. For example, we held a focus group with professionals who worked in small GP practices with more deprived patients. Finally, PPI discussions around difficulties with ‘frailty’ mirrored results from the eFI2 study's data on healthcare professionals’ challenges with explaining frailty to patients who speak English as a second language.

Our PPI process reflected a team that was continuously learning from doing and adjusting elements of the activities to reach diverse voices in more inclusive ways. This is shown in several key points in our process: the switch to London following the Bradford poetry session, recruiting more diverse participants in London following a first group of white well‐educated participants, holding the London activity in a community space, involving participants with limited or no English proficiency during the quilting session, and adjusting the final poetry session to a quilting session. These also reflect a creative PPI approach in which the public collaborators held more power and influence in decision‐making as the project progressed, to the extent that the quilting activity's design was held largely by our public collaborator.

### 3.2 Thematic Reflections

Below, we describe the three thematic reflections, 1) ‘difficulties with ‘frailty’ as a term and concept,’ 2) ‘personalising ‘frailty’ in patient communications,’ and 3) ‘time pressures and dysfunction in healthcare services.’ These reflections were used to help compare what people with lived experience said to professionals in our qualitative study, helping us ensure results were aligned to patient and carer experiences. Quotations have been completely anonymised to protect the identities of participants.

#### 3.2.1 Thematic Reflection 1 Difficulties with ‘Frailty’ As a Term and Concept

Overall, participants heavily critiqued the terms, ‘frailty’ and ‘frail.’ Many participants advised against using them altogether with patients, such as when one Bradford poetry attendee said, ‘Get rid of the word frail!’ One participant even described ‘frailty’ as ‘the road sign for old people.’ Another similarly argued, ‘frailty should not be associated with age, it is an ageless condition.’ It was also discussed that the word does not exist in some languages, such as Urdu, complicating conversations with healthcare professionals.

**Figure 4:**
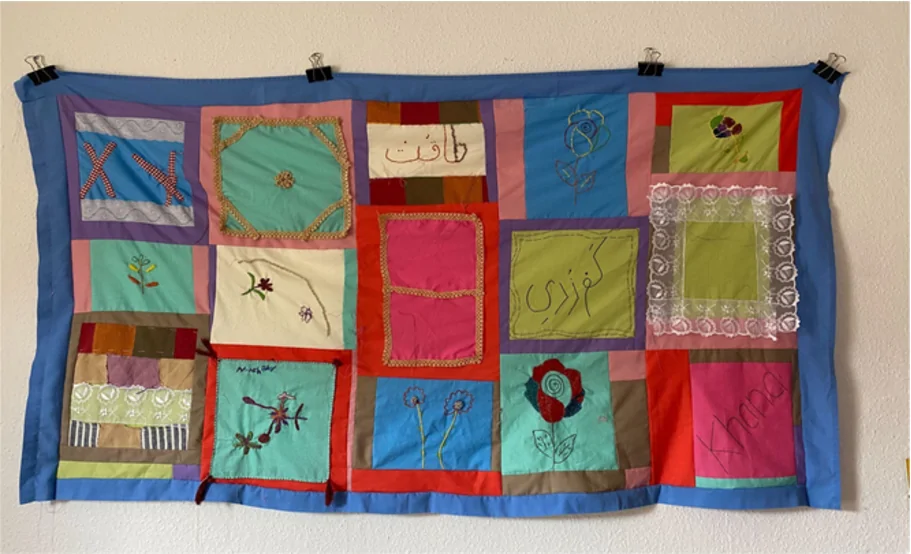
The completed frailty quilt.

This was made even more clear by the difficulties the Bradford quilting participants found in translating ‘frailty’ and ‘frail’ to Urdu. Some of the quilt patches (Figure 4) reflected this challenge and illustrate Urdu words which arose in discussions attempting to find a set of terms relevant to frailty like, ‘*kamzori*,’ (weakness); ‘*khana,*’ (food); and ‘*zhain,*’ (mind).

Others critiqued the concept and scope of ‘frailty,’ saying that it is ‘too big and too broad’ and that it focuses on a medical model understanding of ability, in which frailty is limited to the physical, ignoring isolation and mental health. Other participants emphasised the need to discuss specific symptoms in isolation to prop up the frailty concept, preventing a holistic understanding of a patient's body, a sort of remedy that many found to the flattening and essentializing of ‘frailty.’

Interestingly, participants emphasised that finding a single word to replace ‘frail’ or ‘frailty’ is the wrong approach and that alternative terms, including that which eFI2 research participants suggested, ‘living well,’ brought similar issues as ‘frailty.’ They argued that this alternative term ‘sounds nice, but it could have the wrong connotations [and] lead to unhealthy behaviours’.

#### 3.2.2 Thematic Reflection 2: Personalising ‘Frailty’ in Patient Communications

Attendees described frailty as a ‘hidden subject’, meaning that healthcare professionals usually do not discuss it with patients. Participants observed that when clinicians do discuss frailty with patients, they communicate with relatives rather than the patient themselves. One participant said, ‘I want a GP to talk to me about my conditions, not frailty, [and] about how I can make my life better.’ Another further complicated this, arguing that ‘GPs explain frailty in different ways, which can be confusing.’ This aligned with the results of the eFI2's qualitative data collection with professionals.

While participants strongly disliked the words ‘frail’ and ‘frailty’ and found them unhelpful for patient communications, they recognised that clinicians still used them to understand patients’ health.They desired more transparency around how clinicians understand ‘frailty’ and applied it as a concept in their own healthcare. A London participant summed this up by saying, ‘If a GP is thinking ‘frail, frail, frail,’ but they are not saying that, then it feels deceptive.’ They further said that communications around frailty with patients need to be tangible, practical, and considerate of the ‘whole person,’ for example, using language like, ‘What are your support needs?’ ‘What are your challenges right now?’, and ‘How can we work together?’

Attendees agreed language is important and advised that ‘frailty’ will mean different things to different people. They said it is therefore best to use the patient's own language and understanding to explain what frailty is and how it relates to them. The words they used to describe their health are referenced in the Bradford poem. They further said that communications around frailty with patients need to be tangible, practical, and holistic considering the whole person.

#### 3.2.3 Thematic Reflection 3: Time Pressures and Dysfunction in Health Services

Participants discussed time as one of the greatest hurdles to supporting good frailty communication with professionals. A London poetry participant said, ‘Communication about health conditions feels the best when you have time with a healthcare professional, when you can talk and make sure you are saying the right thing, when you feel as though they are taking time to explain.’

However, participants also described clinicians as frequently pressed for time, For instance, one London participant said, ‘The maximum 10‐min GP appointment and limit of one issue per appointment is frustrating…it feels like ‘they don't want to know.’ It doesn't make sense if you have multiple interconnected issues, and there is no time to have the holistic care that frailty needs…[it] can leave patients feeling unworthy of treatment.’

Some described other issues that overlapped between time, which some described as ‘dysfunctional.’ For example, some participants with lived experience of multiple long‐term health conditions said that getting treatment for long‐term conditions between different clinics and departments ‘can feel like going round in circles, where you feel like you often have to start again [all the time].’ Consequently, the need to advocate for oneself brought about anxieties and experiences around being labelled ‘a problem patient’ by clinicians or staff, particularly worrying that self‐advocacy could negatively impact their healthcare. Another emphasised staff skills and capacity as another contributor to healthcare dysfunction, while finding empathy for them, saying, ‘The workers…haven't been given the tools [they need].’ Finally, other participants attributed the dysfunction in the system to limited funding, citing financial gaps between prevention and treatment.

### 3.3 Reflections on the Poems

We present the Bradford and London poems below. Both poems acknowledge the importance of autonomy in the face of navigating physical changes. Lines in the poem, such as, ‘Hard to negotiate the tension’ echo the thematic reflections above.

#### 3.3.1 ‘Come to My Level’ – the Bradford Poem

Reflecting,

Language that they would use

Not with a patient themselves

Reliability,

I developed my own model

Social decline,

Along the way, that is how you are measured

Like a bathtub curve.

Having frailty is not a moral failing

Get away from it

Not as you, but as the condition

You tried to give and you didn't take

That's how I should be

Aligning to some sort of expectation

In the end we are talking about our reliability

Come to my level.

#### 3.3.2 ‘Worthy of Treatment?’ – the London Poem

It's too big, too broad,

too wide,

The nature of changing.

A tricky course, it undermines

language.

An irreversible state?

Hard to negotiate the tension,

like a round circle

Just ask me

‘How can we work together?’

5 min then the next person is in.

How often am I looked at as

worthy of treatment?

Am I worthy of treatment?

### 3.4 Reflections on the Quilt and Its Image

Some words mentioned in theme 1 around difficulties with frailty, such as ‘*khana,’* are visible in the quilt (Figure 4) and directly relate to terms that the participants’ discussion found useful for thinking about ‘frailty’ in Urdu. However, not all designs easily fit this connection. Other patches in the quilt were not limited to words and were inspired by Pakistani designs significant to the attendees.

The Khidmat Centre community worker observed that the women attending the quilting session, while typically quiet during their regular weekly sessions, were lively chatting amongst each other during the quilting. The quilting attendees, though trepidatious at first about discussing frailty and being involved in research, expressed interest in being involved in future research projects about frailty at the conclusion of the project.

## 4 Discussion

We held three creative PPI workshops in Bradford (n = 2) and London (n = 1). Two were dedicated to poetry (Bradford and London), and one focused on quilt‐making and split over 2 days (Bradford). These workshops yielded 1) adjustments to the eFI2 topic guide and validation of the eFI2 qualitative analysis, 2) three thematic reflections, ‘difficulties with ‘frailty’ as a term and concept,’ ‘personalising ‘frailty’ in patient communications,’ and ‘time pressures and dysfunction in healthcare services;’ and 3) three creative outputs, two poems and a quilt exemplifying patient and carer understandings and critiques of frailty.

### 4.1 Frailty Communication

Harrison et al. have shown that a helpful means of evaluating creative PPI is to consider the impacts or the ‘lessons learned’ that the activities have helped bring about [23]. Overall, the poems, quilt, and themes reflected patients’ and carers’ discomfort with or difficulty defining the terms, ‘frail’ and ‘frailty.’ The creative pieces and their associated themes illustrated pragmatic difficulties with these terms, finding them to be too broad, too vague, or varying in meaning. These lessons chime with current literature, specifically resisting identification as ‘frail’ and avoiding the term ‘frail’ [2, 5, 9]. Some recent work on the frailty concept has attempted to identify means of improving patients’ relationships to it [2, 5]. Our work takes this further by arguing that instead of using ‘frailty’ or a replacement term to describe physical decline, clinicians should talk with patients about their own perceptions and preferred language for talking about their realities and challenges. Hopkins et al. have discussed the practical difficulties involved with replacing ‘frailty’ with a different term from an institutional perspective, as well as identifying a term that effectively ‘minimises negative connotations,’ [2]. Our creative PPI activities show that some patients believe that replacement with another term would likely bring about the same problems, as they find the challenge at the root to be the frailty concept's focus on a medical model understanding of measuring patient decline without considering a more complete person's experiences and subjectivities.

The research team was glad that the PPI workshops aligned with what we had been told in our interviews and focus groups with professionals, confirming that our qualitative study was in keeping with what people with frailty felt. Primarily, we had found that professionals did not use the word ‘frail’ in consultations as they felt patients viewed it negatively. We discussed this as a research team with our advisory group, which helped us to decide to focus on communication of frailty as the topic of a paper which is currently under preparation to be submitted to a peer‐reviewed journal. It will also be the topic of further research in future grant applications, as it was clear that this was an important and sensitive area for people with frailty when they encounter primary care professionals.

#### 4.1.1 ‘Learning As You Go’

These PPI activities show the importance of reflecting on learnings from PPI and then applying changes to the project, or ‘learning as you go’ to conduct successful and meaningful creative PPI [56, 57]. We identified several important moments in which we needed to shift our approach or design to better engage collaborators and to connect with relevant audiences. This included when we pivoted to working with the Khidmat Centre to diversify the voices and when we changed the final activity to a quilting session from a blackout poetry session. While conventional PPI can benefit from this approach, creative PPI should be particularly attuned to the needs and shortcomings of its activities, especially because of the additional logistics and unique demands of creative activities.

### 4.2 Inclusivity, Tokenism, and Accessibility

These creative PPI activities found some evidence for creative PPI's ability to address conventional PPI's gaps around tokenism and accessibility by giving people an alternative to speaking, especially helpful for engaging those labelled as ‘seldom listened to,’ peoples with limited English skills, and people from deprived areas [21, 24, 25, 27]. The activities provoked discussion across Urdu‐English linguistic lines, with the aid of translators, and helped create poems and discussion around patients’ needs for personalised communications. The theme, ‘difficulties with ‘frailty’…’ especially shows support for getting around the challenges of technical language and discussion‐only activities. In our activities’ case, quilting with limited English speakers made it easier to begin talking about frailty than to dive into a conversation about it [20, 21, 23, 24, 27, 28].

### 4.3 Relevance and Applicability

Our creative PPI activities held complex relationships to the benefits and shortcomings of creative PPI as described in the literature [17, 19, 23, 24]. They found some support and some disagreement with the conception of creative PPI outputs as too individually or locally unique to be generalisable. The poems and thematic reflections specifically resonated with critiques of ‘frailty’ as being essentialising and reductionist [2, 4], but other elements, such as the quilt's flower motifs, were more relevant to the women behind its creation than to the frailty concept or the eFI2 study. This is not to downplay the significance of the quilt to these women or the Khidmat Centre, nor its ability to convey and inspire discussions around frailty amongst them. However, insofar as the quilt is embedded within a research process, it is fair to say that the existing research infrastructure does not have a clear way to immediately consider its contributions to research [17].

Harrison et al. have also highlighted considering creative PPI outputs’ applicability as a method of evaluation [23]. The biggest challenge with our creative PPI approaches for the eFI2 was applying the creative pieces and insights into practical changes on the project. Blackout poetry and quilting were excellent approaches for getting members to think about and engage with ideas around frailty while speaking from their perspectives and lived experiences. However, it was extremely difficult to translate a quilt into our qualitative study on clinicians’ use of the eFI2. The activities provided insights into how patients feel about ‘frailty’ and what they would prefer clinicians to say and not to say, some of which were stated directly in the poems themselves. Currently for creative PPI approaches allow people to engage with difficult topics, but care must be given to how these approaches fit into larger projects, to ensure that they contribute to meaningful collaborative research methods. Creative outputs that are not applicable run the risk of acting as science communication or public engagement approaches, instead of the collaborative two‐way involvement that PPI aims to uphold [58].

Perhaps the greatest outcome of these creative PPI workshops transcends relevance and applicability and centres instead on the relationship formed between the facilitators and the Khidmat Centre. Though outside the eFI2 project's scope, the work to create the quilt and its placement at the Centre fuelled a type of collaborative community health support which researchers and health professionals could continue to build on in the future [58].

### 4.4 The Continued Lives of Creative Outputs

A major benefit to creative PPI is that the creative outputs can continue the mission of the research and its related collaboration work. For example, the workshop poems have been shared at an Integrated Care Board (ICB) Frailty Stakeholder event in West Yorkshire by our public collaborator and co‐author. Audience members noted that the poems made discussions on the frailty concept more accessible and engaging to audiences beyond academia [27]. Furthermore, the actual quilt will remain at the Khidmat Centre so that workshop participants can feel ownership over it and that they and other community members can continue to reflect and discuss frailty with other members of the public in this community space, benefits described by Galiatsatos et al. in their work on art as a ‘broker’ of trust in community health [58].

The quilt can also act as a piece of art that still engages with some of the ideas around frailty and provides benefits to patient communities [58, 59, 60, 61, 62]. The quilt's floral patterns and association with comfort can provide a sense of comfort and peace, contrasting with pressures, anxiety, and frustrations that can accompany patients and families experiencing the conditions that cause frailty and accessing health services to treat them. This builds on several recent moves in the NHS to add art to clinics and waiting rooms [63, 64, 65], as well as several research projects’ findings that art in clinical and community spaces can offer health benefits during the healing and recovery processes [58, 59, 60, 61, 62].

### 4.5 Strengths and Limitations

We find the strongest points of this project to be our use of creative PPI and our shifts throughout the project to pursue greater levels of co‐production, diversity, and access throughout the project.

While we did make edits and changes to our eFI2 topic guide and used PPI feedback to validate our analysis of the eFI2 interviews, we did not collect detailed data on what these changes included and are thus unable to share what specific wording changes took place. We advise all studies in the future that are using PPI to improve a topic guide to keep detailed records of how the drafts of topic guides change after PPI activities and how they compare to previous drafts. This can provide clear and strong examples of PPI impacts for and beyond creative approaches.

We also chose not to collect detailed and extensive demographic data on the creative PPI participants. We and our public collaborators were trying to make activities as accessible as possible to attendees and detailed demographic questionnaires can often pose socioemotional burdens on respondents during a first meeting. However, in writing this manuscript, we have noted that the lack of more detailed demographic data of our attendees makes our findings and insights more difficult to apply and generalise to others. We recommend using inclusive demographics questions to have clear and detailed descriptions of those involved in activities to facilitate the best understanding of their context as possible [60].

Our reliance on pre‐existing PPI networks and community gatekeepers may have introduced selection bias into the recruitment process. Workshop participants may not represent broader populations living with frailty, particularly those with severe cognitive or physical impairments or those with limited experiences with PPI and research.

Given the weaknesses of our creative PPI activities, this paper offers unique and helpful examples of creative methodologies, patient views on frailty, and insights into communication approaches for clinicians and researchers around frailty. Importantly, limited English language abilities is a chief barrier to health and research access for peoples in the UK [66, 67]. Though it is possible that translator and facilitator bias was introduced in the quilting session, our multilingual approach to dealing with an English language barrier in PPI and health research represents a unique and helpful example, if imperfect.

Finally, though small by some comparisons (n = 22), the number of participants in our creative PPI activities is common for published examples of PPI activities [68, 69, 70, 71].

## 5 Conclusion

Creative PPI can help create space to discuss and understand the nuances and needs that patients have around discussing frailty. Communication between patients and clinicians around frailty will likely continue to encounter challenges, but using approaches like creative PPI to explore communication options can centre on patients and improve these conversations. Research and healthcare professionals should work towards discussing frailty with patients in terms and concepts that reflect their experiences and understanding and consider them more fully, including their mental health. Future research and PPI should focus on diverse cultural perspectives on frailty, especially in cultures and languages where no such concept exists. It should also focus on the challenges around applying insights from creative PPI outputs and what their clear impacts are.

## Author Contributions

**William Lammons:** conceptualisation, methodology, formal analysis, writing – review and editing, writing – original draft. **Lily Arnold:** conceptualisation, funding acquisition, writing – original draft, methodology, validation; writing – review and editing, formal analysis, project administration. **Sarah Mcmullen:** conceptualisation, methodology, project administration, supervision. **Fiona Aspinal:** funding acquisition, conceptualisation, investigation, methodology, validation, writing – review and editing, project administration, supervision. **Samina Begum:** conceptualisation, methodology, writing – review and editing, project administration, resources, funding acquisition, writing – original draft. **Katherine Barrett:** writing – review and editing, methodology, writing – original draft, project administration. **Kate Walters:** conceptualisation, supervision, funding acquisition, investigation, validation. **Andrew Clegg:** investigation, conceptualisation, funding acquisition, validation, supervision. **Stuart Mackay‐Thomas:** conceptualisation, investigation, funding acquisition, supervision, validation. **Sara Humphrey:** conceptualisation, investigation, funding acquisition, writing – original draft, methodology, validation, project administration, supervision. **Danielle Nimmons:** conceptualisation, investigation, funding acquisition, writing – original draft, writing – review and editing, validation, methodology, formal analysis, project administration, supervision, resources.

## Ethics Statement

The study's ethical approval number is 24/HRA/2407. The study's data protection number is Z6364106/2023/11/127.Two public collaborators (SB, KB) worked throughout the process of delivering, analysing, and writing‐up the creative PPI workshops. They helped the team improve diversity and access to PPI activities.

## Conflicts of Interest

The authors declare no conflicts of interest.

## Supporting information


Supporting File

## Data Availability

Research data are not shared. All relevant data are included in the manuscript.
