# Supplementary material for: Group Poetry and Quilt‐Making –Creative Public Involvement Approaches to Contentious Concepts in Frailty Research
Source: Health Expect. 2026 Sep 14;29(5):e70872. doi: 10.1111/hex.70872 (PMC13573230; doi:10.1111/hex.70872)
Supplement: Supplementary file 1 [file HEX-29-e70872-s001.docx]

Supplement 1 – Prep poem, Frailty is Just a Word

Jackie Hardy, wrote the following poem in her own time in anticipation of the poetry session. She shared this with us and allowed us to publish it here as part of the manuscript.

**Frailty is Just a Word**

*Jackie Hardy*

I may be frail and fall

But I get up and keep going

I may be getting older but I should not be treated as invisible

Too many doctor and hospital appointments, I just want a break

Let's keep going, you've got this

We believe in ourselves, so let's keep living and laughing and try not to pee in the process

Frailty is just a word and we are so much more. Let's celebrate us and our uniqueness. We are special people and have so much to offer

Supplement 2 – GRIPP2

| Section and Topic | Item | Page | | |
| --- | --- | --- | --- | --- |
| 1a. Aims | Report the aim of the study relating to PPI | 5 | | |
| 1b. Methods | Describe the PPI methods used in the study | 5-14 | | |
| 1c. Results | Report the impacts and outcomes of PPI in the study | 15-19 | | |
| 1d. Conclusions | Summarise the main conclusions relating to PPI in the study | 22-23 | | |
| 1e. Keywords | Include PPI, ‘patient and public involvement’ or alternative terms as keywords | 2-3 | | |
| 2a Definition | Report the definition of PPI used in the study, including any rationale | 4 | | |
| 2b. Definition | Report how your definition links to those provided by other comparable studies | 4-5 | | |
| 2c. Concepts and theory development | Report the way in which PPI is being conceptualised | 4-5 | | |
| 2d. Concepts and theory development | Report any conceptual or theoretical models, or influences, used in the study | 4-5 | | |
| 3. Aims | Report the aim of PPI in the study | 5 | | |
| 4a. Design | Provide a clear description of methods used for PPI in the study | 8-14 | | |
| 4b. Population | Provide a description of patients, carers and the public involved with the PPI activity in the study | 8-9 | | |
| 4c Stages of involvement | Report on how PPI is used at different stages of the study (e.g. Identifying and prioritising research ideas, designing and managing research, undertaking the research, and dissemination and implementation of research) | 5 | | |
| 4d. Level or nature of involvement | Report the level or nature of PPI used at various stages of the study (e.g. some people aim for a collaborative approach) | 7 | |  |
| 5a. Qualitative evidence of impact | If applicable, report the method used to qualitatively capture the impact of PPI in the study | 20-22 | |  |
| 5b. Quantitative evidence of impact | If applicable, report the method used to quantitatively measure or assess the impact of PPI | n/a | |  |
| 5c. Robustness of measure | If applicable, report the robustness of the method used to capture or measure the impact of PPI | n/a | |  |
| 6. Economic Assessment | If applicable, report the method used for an economic assessment of PPI | n/a | |  |
| 7a. Outcomes of PPI | Report the results of PPI in the study, including both positive and negative outcomes | 20-22 |  |  |
| 7b. Impacts of PPI | Report the specific impacts that PPI has had on the research, the individuals involved (including patients and researchers), and on communities, including both positive and negative impacts | 20-22 |  |  |
| 7c. Context of PPI | Report the influence of any contextual factors (e.g. availability of funding, policy) that enabled or hindered the impact of PPI | 8-9 |  |  |
| 7d. Process of PPI | Report the influence of any process factors (e.g. how users were involved), that enabled or hindered the impact of PPI | 8-14 |  |  |
| 7e. Concept and theory development | i) Report any key conceptual or theoretical developments in PPI that have emerged from the study | 20-22 |  |  |
|  | ii) If applicable, report any testing of conceptual or theoretical models | n/a |  |  |
| 7f Measurement | If applicable, report all aspects of instrument development and testing (e.g. validity, reliability, feasibility, acceptability, responsiveness, interpretability, appropriateness, precision) | n/a |  |  |
| 7g. Economic assessment | Report any information on the economic cost or benefit of PPI | n/a |  |  |
| 8a. Outcomes | Comment on the extent to which PPI influenced the study overall. Describe positive and negative effects | 20-22 |  |  |
| 8b. Impacts | Comment on the different impacts identified in this study and how they contribute to the new knowledge | 20-22 |  |  |
| 8c. Definition | Comment on the definition of PPI used in the study and whether you would suggest any changes | 4-5 |  |  |
| 8d. Conceptual development and theory development | Comment on how this study adds to conceptual or theoretical development of PPI | 20-22 |  |  |
| 8e. Context and process | Comment on how context and process factors influenced PPI in the study | 22 |  |  |
| 8f. Measurement and capture of PPI impact | If applicable, comment on how well PPI impact was captured or measured in the study | n/a |  |  |
| 8g. Economic information | If applicable, discuss any aspects of economic cost or benefit of PPI, particularly any suggestions for future economic modelling. | n/a |  |  |
| 8h. Critical perspective | Comment critically on the study, reflecting on the things that went well and those that did not, so others can learn from this experience | 22 |  |  |
